# Diagnostic Accuracy of Parameters for Zika and Dengue Virus Infections, Singapore

**DOI:** 10.3201/eid2312.171224

**Published:** 2017-12

**Authors:** Hanley J. Ho, Joshua G.X. Wong, Win Mar Kyaw, David C. Lye, Yee Sin Leo, Angela Chow

**Affiliations:** Tan Tock Seng Hospital, Singapore

**Keywords:** disease outbreaks, dengue, mosquitoes, Aedes, Zika virus, dengue virus, viruses, vector-borne infections, Singapore

## Abstract

Singapore experienced its first documented Zika virus outbreak in 2016. We identified clinical and laboratory parameters that increase the probability for Zika or dengue virus infection. Early during the illness, combinations of key parameters obtained through clinical assessment and hematologic tests can help distinguish between these infections.

Zika virus recently emerged as a clinically important arbovirus that can cause fetal complications in infected pregnant women and Guillain-Barré syndrome in adults (*1**–**3*). Since the first reported large Zika outbreak on Yap Island in 2007 (*4*), widespread community outbreaks have been reported in many other countries (*4**,**5*).

Dengue virus (DENV) threatens the 50% of the world’s population who live in at-risk areas and causes ≈390 million infections annually (*6*). The frequency and magnitude of epidemic dengue have increased exponentially during the past 4 decades because of such factors as population growth and rising temperatures (*7*). Severe dengue can result in plasma leakage, systemic shock, multiorgan failure, and eventual death (*8*).

Zika and dengue have similar presenting symptoms (including fever, rash, arthralgia, myalgia, and headache) (*4**,**9*); incubation periods; and transmission routes through *Aedes* mosquitos (*5**,**6*). Accurate and early diagnosis is essential to properly manage the unique complications of each disease.

In Singapore, a tropical island city-state in Southeast Asia, DENV is endemic. Dengue epidemics have been recorded every few years since the 1990s and now predominantly affect adults (*8*). In August 2016, Singapore experienced its first documented Zika outbreak (*10*). Before that outbreak, national surveillance of community-based patients (ongoing since 2014) had not detected any local Zika cases (*11*). The Zika outbreak occurred on the background of ongoing DENV circulation, and medical practitioners had to consider concurrent testing for both infections as part of clinical management.

Using the likelihood ratio approach, we identified clinical and laboratory parameters that increase the likelihood of a laboratory-confirmed diagnosis of Zika virus or DENV infection at first presentation to clinical care. The clinical diagnostic process may lack sensitivity and specificity (*12*). A positive likelihood ratio (LR+) is calculated using the proportion of patients with the disease having a positive clinical or laboratory finding divided by the proportion of patients without the disease having that same finding (*13*). This information adds value to clinical diagnosis by refining the posttest probability of a disease. In the absence of confirmatory laboratory tests, a thorough assessment of such parameters may help distinguish between the 2 diseases.

## The Study

We reviewed 2 prospectively recruited cohorts of patients with suspected Zika virus and DENV infections treated at Tan Tock Seng Hospital (Singapore), an adult tertiary care hospital. This hospital also houses the Communicable Disease Centre, the designated institution for centralized management of emerging infectious diseases in Singapore.

The Zika cohort comprised persons with suspected Zika virus infection recruited during August and September 2016. We followed the case definition used by the Singapore Ministry of Health (i.e., any person living, working, or studying in the outbreak area with fever and maculopapular rash plus >1 additional symptom of arthralgia, myalgia, headache, or conjunctivitis). Patients whose illness partially or fully met the case definition underwent confirmatory laboratory testing through detection of Zika virus RNA in serum and urine samples using real-time reverse transcription PCR (RT-PCR) (*14*). Confirmed cases were defined as a positive result for a Zika virus serum or urine test. We excluded patients with laboratory-confirmed dengue co-infection, using dengue NS1 antigen (Bio-Rad Laboratories, Marnes-la-Coquette, France) or RT-PCR (*15*).

The dengue cohort comprised persons with suspected DENV infection recruited during January 2010–September 2012. Persons in this cohort had fever (temperature >37.5°C), with or without additional signs or symptoms, and no alternative diagnosis at the time they sought care. Cases were defined as a serum-positive DENV NS1 antigen or RT-PCR.

To evaluate parameters during the early phase of each illness for both cohorts, we limited participant recruitment to persons who sought care within 5 days after symptom onset. We obtained ethics approval from the National Healthcare Group (NHG Domain Specific Review Board reference no. 2016/01027).

The Zika cohort comprised 281 persons with suspected Zika virus infection (Technical Appendix Table 1): 130 case-patients (without dengue co-infection) and 151 non–case-patients. The median age of case-patients (34 years [interquartile range (IQR) 26–49 years]) was similar to that of non–case-patients (31 years [IQR 24–38 years]). Sex distribution was similar between case-patients (60% male) and non–case-patients.

**Table 1:** Diagnostic accuracy of clinical and laboratory parameters for Zika virus infection cohort. Singapore*

Parameters	Sensitivity (95% CI)	Specificity (95% CI)	PPV (95% CI)	NPV (95% CI)	LRP (95% CI)	LRN (95% CI)
Clinical						
Documented fever >37.5°C	0.35 (0.26–0.43)	0.58 (0.50–0.66)	0.42 (0.32–0.51)	0.51 (0.43–0.58)	0.83 (0.61–1.12)	1.12 (0.93–1.35)
Headache	0.23 (0.16–0.30)	0.62 (0.54–0.69)	0.34 (0.24–0.44)	0.48 (0.41–0.55)	0.60 (0.41–0.87)	1.25 (1.07–1.46)
Myalgia	0.41 (0.33–0.49)	0.41 (0.34–0.47)	0.32 (0.25–0.39)	0.5 (0.42–0.57)	0.66 (0.52–0.84)	1.54 (1.20–1.97)
Arthralgia	0.21 (0.14–0.28)	0.79 (0.72–0.85)	0.46 (0.33–0.58)	0.54 (0.47–0.60)	0.98 (0.62–1.54)	1.01 (0.90–1.13)
Rash	0.94 (0.90–0.98)	0.59 (0.51–0.67)	0.67 (0.59–0.73)	0.92 (0.86–0.97)	2.29 (1.88–2.78)	0.10 (0.05–0.21)
Conjunctivitis	0.23 (0.16–0.3)	0.88 (0.83–0.93)	0.63 (0.49–0.76)	0.57 (0.51–0.63)	1.93 (1.13–3.31)	0.87 (0.78–0.98)
Nausea	0.04 (0.01–0.07)	0.95 (0.91–0.98)	0.38 (0.12–0.65)	0.53 (0.47–0.59)	0.73 (0.24–2.16)	1.02 (0.96–1.07)
Vomiting	0.02 (0–0.04)	0.94 (0.90–0.98)	0.18 (0–0.41)	0.53 (0.47–0.58)	0.26 (0.06–1.17)	1.04 (1.00–1.10)
Diarrhea	0.03 (0.001–0.06)	0.97 (0.94–1.00)	0.44 (0.12–0.77)	0.54 (0.48–0.60)	0.93 (0.25–3.39)	1.00 (0.96–1.05)
Abdominal pain	0.02 (0–0.04)	0.98 (0.96–1.00)	0.40 (0–0.82)	0.54 (0.48–0.60)	0.77 (0.13–.57)	1.00 (0.97–1.04)
Runny nose	0.02 (0–0.05)	0.92 (0.88–0.96)	0.20 (0–0.40)	0.52 (0.46–0.58)	0.29 (0.08–1.01)	1.06 (1.006–1.12)
Sore throat	0.18 (0.12–0.25)	0.83 (0.77–0.89)	0.48 (0.34–0.62)	0.54 (0.48–0.61)	1.07 (0.65–1.77)	0.98 (0.88–1.10)
Cough	0.12 (0.06–0.17)	0.72 (0.64–0.79)	0.26 (0.15–0.37)	0.49 (0.42–0.55)	0.41 (0.24–0.69)	1.24 (1.10–1.39)
Breathlessness	0.01 (0–0.02)	0.97 (0.95–1.00)	0.20 (0–0.55)	0.53 (0.47–0.59)	0.29 (0.03–2.56)	1.02 (0.99–1.05)
						
Laboratory†						
Leukocytes <3.6 × 10^9^ cells/L	0.17 (0.11–0.24)	0.9 (0.85–0.94)	0.54 (0.4–0.69)	0.6 (0.55–0.66)	2.00 (1.06–3.79)	0.90 (0.82–0.99)
Hemoglobin <13 g/dL	0.22(0.15–0.29)	0.80 (0.74–0.87)	0.50 (0.37–0.63)	0.54 (0.47–0.60)	1.13 (0.72–1.79)	0.97 (0.86–1.09)
Platelets <100 × 10^9^/L	0.01 (0–0.02)	0.99 (0.97–1.00)	0.33 (0–0.87)	0.53 (0.47–0.59)	0.57 (0.05–6.16)	1.01 (0.98–1.03)
Neutrophils <1.4 × 10^9^ cells/L	0.06 (0.02–0.10)	0.99 (0.97–1.00)	0.80 (0.55–1.00)	0.53 (0.47–0.59)	4.27 (0.93–19.78)	0.95 (0.91–1.00)
Neutrophils >5.9 × 10^9^ cells/L	0.04 (0.01–0.08)	0.66 (0.58–0.74)	0.1 (0.02–0.18)	0.42 (0.36–0.49)	0.11 (0.05–0.28)	1.45 (1.28–1.64)
Lymphocytes <0.9 × 10^9^ cells/L	0.18 (0.12–0.25)	0.78 (0.71–0.85)	0.44 (0.31–0.57)	0.50 (0.44–0.58)	0.83 (0.51–1.33)	1.04 (0.93–1.18)
Creatinine >105 μmol/L	0.08 (0.03–0.13)	0.94 (0.91–0.98)	0.56 (0.33–0.79)	0.54 (0.47–0.60)	1.41 (0.57–3.46)	0.98 (0.91–1.04)
Urea >9.3 mmol/L	0	0.99 (0.98–1.00)	0	0.54 (0.48–0.59)	0	1.01 (0.99–1.02)
Alanine aminotransferase >54 U/L	0.08 (0.03–0.13)	0.94 (0.91–0.98)	0.56 (0.33–0.79)	0.56 (0.5–0.61)	0.72 (0.37–1.39)	1.05 (0.96–1.14)
Aspartate aminotransferase >41 U/L	0.07 (0.03–0.11)	0.83 (0.77–0.89)	0.27 (0.12–0.42)	0.50 (0.43–0.56)	0.42 (0.20–0.86)	1.12 (1.02–1.22)
						
Combinations						
Any GI symptom (nausea, vomiting, diarrhea, abdominal pain)	0.06 (0.02–0.10)	0.89 (0.84–0.94)	0.33 (0.14–0.52)	0.53 (0.46–0.59)	0.58 (0.26–1.31)	1.04 (0.98–1.13)
Any GI symptom + lymphopenia	0.01 (0–0.03)	0.96 (0.93–0.99)	0.20 (0–0.45)	0.56 (0.5–0.61)	0.53 (0.10–2.87)	1.01 (0.98–1.05)
Rash + conjunctivitis	0.22 (0.15–0.29)	0.97 (0.94–0.99)	0.85 (0.73–0.97)	0.59 (0.53–0.65)	6.73 (2.68–16.90)	0.80 (0.73–0.89)
Documented fever + rash	0.31 (0.23–0.39)	0.86 (0.81–0.92)	0.66 (0.54–0.77)	0.59 (0.53–0.66)	2.21 (1.38–3.55)	0.80 (0.71–0.92)
Documented fever + rash + any GI symptom	0.02 (0–0.04)	0.99 (0.98–1.00)	0.75 (0.33–1.17)	0.54 (0.48–0.60)	3.48 (0.37–33.10)	0.98 (0.95–1.01)
Documented fever + rash + lymphopenia	0.07 (0.02–0.11)	0.94 (0.90–0.98)	0.50 (0.27–0.73)	0.53 (0.47–0.60)	1.14 (0.47–2.78)	0.99 (0.93–1.05)
Documented fever + lymphopenia	0.10 (0.05–0.15)	0.86 (0.80–0.91)	0.38 (0.22–0.55)	0.52 (0.46–0.58)	0.70 (0.37–1.34)	1.05 (0.96–1.15)
Documented fever + thrombocytopenia	0	0.98 (0.97–1.00)	0	0.53 (0.48–0.59)	0	1.01 (0.99–1.03)
Documented fever + lymphopenia + thrombocytopenia	0	0.99 (0.97–1.00)	0	0.53 (0.48–0.59)	0	1.01 (0.99–1.03)
Documented fever + rash, with >1 of arthralgia, myalgia, headache, conjunctivitis	0.21 (0.14–0.28)	0.89 (0.84–0.94)	0.63 (0.48–0.77)	0.57 (0.50–0.63)	1.96 (1.11–3.47)	0.90 (0.80–0.98)

*GI, gastrointestinal; LRN, negative likelihood ratio; LRP, positive likelihood ratio; NPV, negative predictive value; PPV, positive predictive value. †Reference values: leukocytes, 3.6–9.3 × 10^9^ cells/L; hemoglobin, 11.0–15.0 g/dL; platelets, 170–420 × 10^9^/L; neutrophils, 1.40–5.90 × 10^9^ cells/L; lymphocytes, 0.90–3.30 × 10^9^ cells/L; creatinine, 40–75 μmol/L; urea, 2.9–9.3 mmol/L; alanine aminotransferase, 14–54 U/L; aspartate aminotransferase, 15–41 U/L.

The DENV cohort comprised 310 persons with suspected DENV infection: 175 case-patients and 135 non–case-patients. Age groups were similar for case-patients (median age 36 years [IQR 29–43 years]) and non–case-patients (median age 32 years [IQR 27–42 years]), and both groups consisted primarily of male patients.

**Figure 1:**
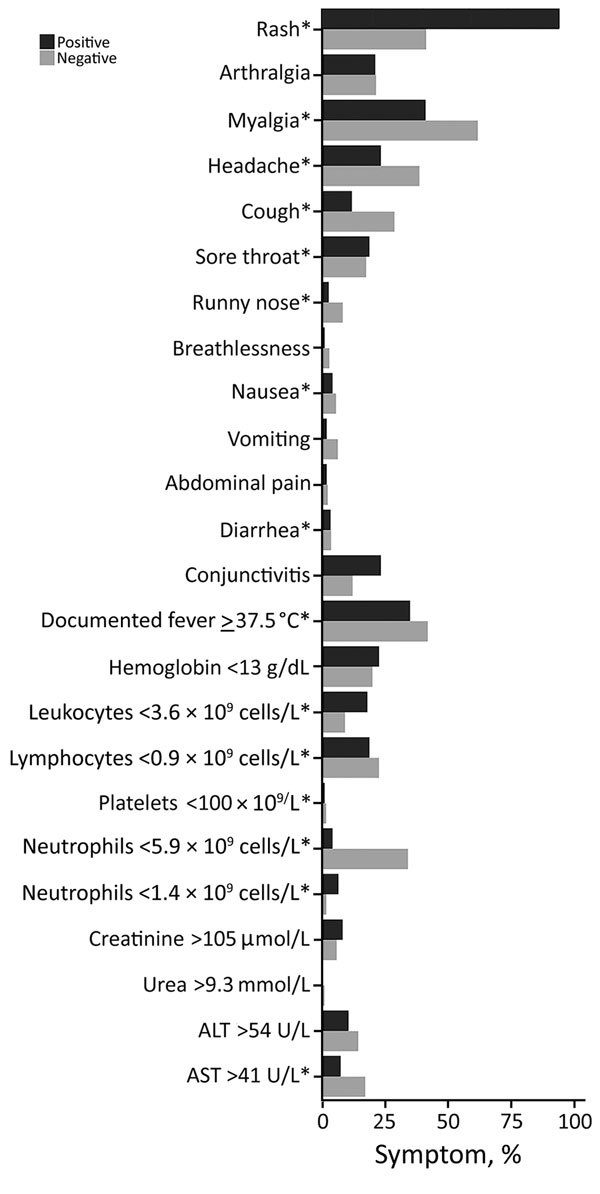
Clinical and laboratory parameters of Zika virus cohort, Singapore. *Statistically significant differences (p<0.05).

Zika virus infection case-patients most commonly had rash (94%), myalgia (41%), and documented fever (35%) (Figure 1); non–case-patients mainly had myalgia (62%), documented fever (42%), and rash (41%). Low proportions of both groups had marked thrombocytopenia (platelets <100 × 10^9^/L [reference 170–420 × 10^9^/L]), leukopenia, or lymphopenia.

**Figure 2:**
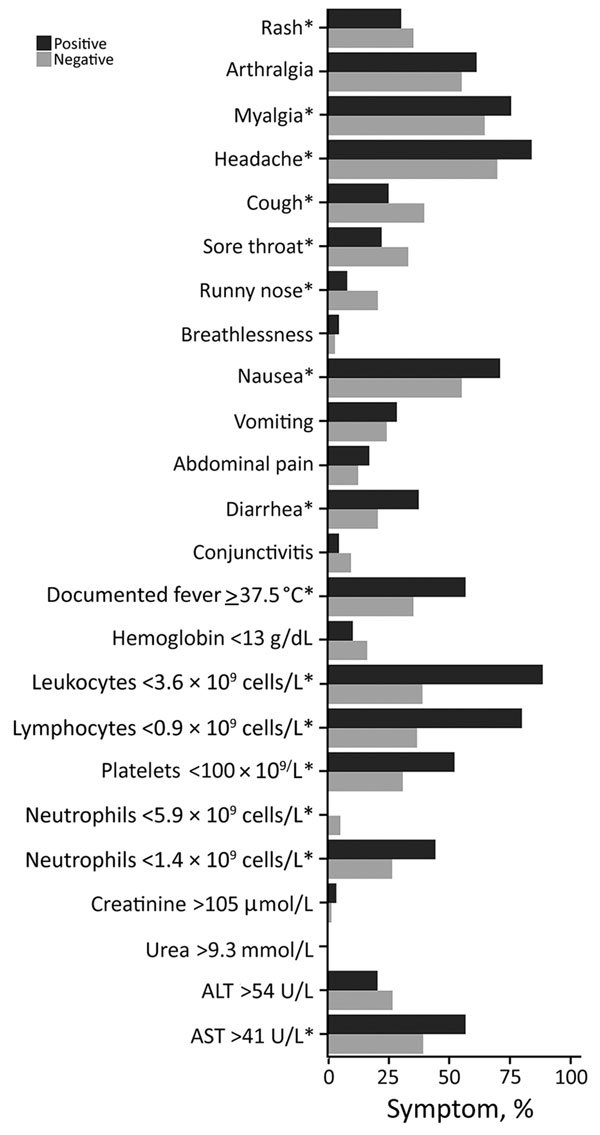
Clinical and laboratory parameters of dengue cohort, Singapore. *Statistically significant differences (p<0.05).

DENV case-patients and non–case-patients most commonly had headache, myalgia, and nausea (Figure 2). Eighty-two percent of case-patients reported gastrointestinal symptoms and, compared with non–case-patients, much higher proportions of leukopenia (89% vs. 39%), lymphopenia (81% vs. 37%), and marked thrombocytopenia (53% vs. 31%).

For assessment of Zika virus infection, among individual parameters evaluated, presence of rash gave the highest LR+, sensitivity and negative predictive value, and lowest negative likelihood ratio (Table 1). We obtained the highest LR+ using a combination of rash and conjunctivitis (LR+ = 6.73, 95% CI 2.68–16.90).

For assessment of DENV infection, documented fever gave the highest individual LR+ of 3.13 (95% CI 2.48–3.94) (Table 2). We obtained the highest LR+ using a combination of documented fever, lymphopenia, and thrombocytopenia (LR+ = 5.11, 95% CI 2.51–10.38).

**Table 2:** Diagnostic accuracy of clinical and laboratory parameters for dengue virus infection cohort, Singapore*

Parameter	Sensitivity (95% CI)	Specificity (95% CI)	PPV (95% CI)	NPV (95% CI)	LRP (95% CI)	LRN (95% CI)
Clinical						
Documented fever >37.5°C	0.57 (0.50–0.64)	0.64 (0.56–0.73)	0.68 (0.60–0.75)	0.54 (0.46–0.61)	3.13 (2.48–3.94)	0.61 (0.54–0.69)
Headache	0.84 (0.79–0.90)	0.30 (0.22–0.37)	0.61 (0.55–0.67)	0.60 (0.48–0.71)	1.20 (1.06–1.36)	0.52 (0.34–0.80)
Myalgia	0.76 (0.70–0.82)	0.35 (0.27–0.43)	0.6 (0.54–0.67)	0.53 (0.42–0.63)	1.17 (1.005–1.35)	0.69 (0.49–0.98)
Arthralgia	0.62 (0.55–0.69)	0.44 (0.36–0.53)	0.59 (0.52–0.66)	0.47 (0.36–0.56)	1.11 (0.92–1.34)	0.86 (0.66–1.12)
Rash	0.30 (0.23–0.37)	0.64 (0.56–0.73)	0.52 (0.43–0.62)	0.42 (0.35–0.48)	0.85 (0.62–1.17)	1.08 (0.92- 1.27)
Conjunctivitis	0.05 (0.01–0.08)	0.90 (0.85–0.95)	0.38 (0.17–0.59)	0.42 (0.37–0.48)	0.47 (0.20–1.11)	1.06 (0.99–1.13)
Nausea	0.71 (0.65–0.78)	0.44 (0.36–0.53)	0.63 (0.56–0.69)	0.54 (0.45–0.64)	1.29 (1.08–1.54)	0.64 (0.48–0.87)
Vomiting	0.29 (0.22–0.35)	0.76 (0.68–0.83)	0.60 (0.50–0.71)	0.45 (0.38–0.51)	1.17 (0.80–1.71)	0.94 (0.83–1.08)
Diarrhea	0.38 (0.31–0.45)	0.79 (0.72–0.86)	0.70 (0.61–0.79)	0.50 (0.43–0.56)	1.82 (1.24–2.66)	0.79 (0.68–0.91)
Abdominal pain	0.17 (0.12–0.23)	0.87 (0.82–0.93)	0.64 (0.50–0.78)	0.45 (0.39–0.51)	1.36 (0.78–2.36)	0.95 (0.86–1.04)
Runny nose	0.08 (0.04–0.12)	0.79 (0.72–0.86)	0.33 (0.19- 0.48)	0.40 (0.34- 0.46)	0.39 (0.21–0.70)	1.16 (1.05–1.28)
Sore throat	0.22 (0.16–0.28)	0.67 (0.59–0.75)	0.46 (0.36- 0.57)	0.40 (0.33–0.46)	0.67 (0.46–0.96)	1.17 (1.01–1.35)
Cough	0.25 (0.19–0.32)	0.60 (0.52–0.68)	0.45 (0.35–0.55)	0.38 (0.32–0.45)	0.63 (0.45–0.87)	1.25 (1.06–1.47)
Breathlessness	0.05 (0.01–0.08)	0.97 (0.94–1.00)	0.67 (0.40–0.93)	0.44 (0.38–0.50)	1.54 (0.47–5.02)	0.98 (0.94–1.03)
Laboratory†						
Leukocytes <3.6 × 10^9^ cells/L	0.89 (0.85–0.94)	0.61 (0.53–0.69)	0.75 (0.69–0.81)	0.82 (0.74–0.89)	2.27 (1.83–2.82)	0.18 (0.11–0.28)
Hemoglobin <13 g/dL	0.10 (0.06–0.15)	0.84 (0.77–0.90)	0.45 (0.30–0.60)	0.42 (0.36–0.48)	0.63 (0.35–1.12)	1.07 (0.98–1.17)
Platelets <100 × 10^9^/L	0.53 (0.45–0.60)	0.67 (0.59–0.75)	0.67 (0.59–0.75)	0.52 (0.45–0.59)	1.58 (1.20–2.08)	0.71 (0.58–0.87)
Neutrophils <1.4 × 10^9^ cells/L	0.45 (0.37–0.52)	0.73 (0.66–0.81)	0.68 (0.60–0.77)	0.51 (0.44–0.58)	1.67 (1.21–2.31)	0.76 (0.64–0.89)
Neutrophils >5.9 × 10^9^ cells/L	0	0.95 (0.91–0.99)	0	0.42 (0.37–0.48)	0	1.05 (1.01–1.10)
Lymphocytes <0.9 × 10^9^ cells/L	0.81 (0.75–0.86)	0.63 (0.55–0.71)	0.74 (0.68–0.80)	0.71 (0.63–0.80)	2.17 (1.73–2.74)	0.31 (0.22–0.43)
Creatinine >105 μmol/L	0.03 (0.01–0.06)	0.98 (0.97–1.00)	0.75 (0.45–1.00)	0.44 (0.38–0.50)	2.31 (0.47–11.39)	0.98 (0.95–1.01)
Alanine aminotransferase >54 U/L	0.21 (0.15–0.27)	0.73 (0.66–0.81)	0.50 (0.38–0.62)	0.41 (0.35–0.47)	0.77 (0.51–1.15)	1.09 (0.96–1.23)
Aspartate aminotransferase >41 U/L	0.57 (0.50–0.64)	0.60 (0.52–0.69)	0.65 (0.57- 0.73)	0.52 (0.44–0.60)	1.44 (1.13–1.85)	0.71 (0.57–0.88)
Combinations						
Any GI symptom (nausea, vomiting, diarrhea, abdominal pain)	0.82 (0.77–0.88)	0.37 (0.29–0.45)	0.63 (0.57–0.69)	0.62 (0.51–0.72)	1.31 (1.13–1.51)	0.48 (0.32–0.70)
Any GI symptom + lymphopenia	0.66 (0.59–0.73)	0.76 (0.68–0.83)	0.78 (0.71–0.85)	0.63 (0.56–0.71)	2.71 (1.98–3.72)	0.45 (0.35–0.56)
Rash + conjunctivitis	0.02 (0.01–0.04)	0.93 (0.89–0.98)	0.31 (0.06–0.56)	0.42 (0.37–0.48)	0.34 (0.11–1.09)	1.04 (0.99–1.10)
Documented fever + rash	0.15 (0.10–0.20)	0.92 (0.87–0.96)	0.70 (0.56–0.85)	0.45 (0.40–0.51)	1.82 (0.93–3.55)	0.93 (0.86–1.00)
Documented fever + rash + any GI symptom + lymphopenia	0.01 (0.06–0.15)	0.96 (0.92–0.99)	0.76 (0.59–0.93)	0.45 (0.39–0.51)	2.44 (1.003–5.95)	0.93 (0.88–0.99)
Documented fever + rash + any GI symptom	0.14 (0.09–0.19)	0.96 (0.92–0.99)	0.80 (0.66–0.94)	0.46 (0.40–0.52)	3.09 (1.30–7.34)	0.90 (0.84–0.97)
Documented fever + rash + lymphopenia	0.12 (0.07–0.17)	0.94 (0.90–0.98)	0.72 (0.56–0.89)	0.45 (0.39–0.51)	2.03 (0.93–4.40)	0.94 (0.87–1.00)
Documented fever + lymphopenia	0.51 (0.44–0.59)	0.84 (0.77–0.90)	0.80 (0.73–0.88)	0.57 (0.50–0.64)	3.16 (2.10–4.75)	0.58 (0.50–0.69)
Documented fever + thrombocytopenia	0.33 (0.26–0.40)	0.88 (0.83–0.94)	0.78 (0.69–0.88)	0.50 (0.44–0.57)	2.74 (1.66–4.56)	0.76 (0.68–0.86)
Documented fever + lymphopenia + thrombocytopenia	0.30 (0.23–0.37)	0.94 (0.90–0.98)	0.87 (0.78–0.95)	0.51 (0.45–0.57)	5.11 (2.51–10.38)	0.74 (0.67–0.82)
Documented fever + rash, with >1 of arthralgia, myalgia, headache, conjunctivitis	0.14 (0.09–0.19)	0.93 (0.89–0.98)	0.74 (0.59–0.88)	0.73 (0.40–0.52)	2.14 (1.03–4.43)	0.92 (0.85–0.99)

*GI, gastrointestinal; LRN, negative likelihood ratio; LRP, positive likelihood ratio; NPV, negative predictive value; PPV, positive predictive value. †Reference values: leukocytes, 3.6–9.3 × 10^9^/L; hemoglobin, 11.0–15.0 g/dL; platelets, 170–420 ×10^9^/L); neutrophils, 1.40–5.90 × 10^9^/L; lymphocytes, 0.90–3.30 × 10^9^/L; creatinine, 40–75 μmol/L; urea, 2.9–9.3 mmol/L;alanine aminotransferase, 14–54 U/L; aspartate aminotransferase, 15–41 U/L.

## Conclusions

Our study demonstrated some key differences between the 2 diseases. Presence of rash featured much more prominently in Zika virus infection than DENV infection during the first 5 days of illness. For dengue patients, rashes usually appear during the critical or recovery phases (typically around the fifth day of illness or thereafter) (*9*). Also, in contrast with dengue patients, relatively few Zika patients had hematologic abnormalities.

Regardless of the patient’s pretest probability for a disease, the change in posttest probability is approximated by a constant (*13*). In our study, presence of rash with conjunctivitis gave the highest increase in probability (≈40%) of Zika virus infection, whereas a combination of documented fever, lymphopenia, and thrombocytopenia increased the probability for DENV infection by ≈30% (Technical Appendix Table 2). In contrast, absence of rash in a patient with suspected Zika or absence of lymphopenia in a patient with suspected dengue reduced the probability of the respective disease by 30%–45%. In countries where these viruses co-circulate, and where access to confirmatory laboratory testing is limited, these may be simple methods to help medical practitioners assess a patient suspected to have Zika or dengue.

The strengths of our study include the analysis of common parameters, standardized to ensure comparability across the 2 cohorts. Our analysis presents likelihood ratios, which are easy to interpret and aid clinical diagnosis by refining the posttest probability of a disease. The main study limitation is that our analysis compared 2 cohorts recruited at separate times and hence does not directly distinguish between the 2 infections. However, hospital workflows and clinical assessment methods for patients largely did not change between these periods. In both cohorts, cases could have been misclassified as noncases because RT-PCR might have missed patients who sought care late. We reduced this risk by restricting recruitment to patients seeking care within 5 days after symptom onset. Our study demonstrates that a thorough assessment of clinical and hematologic parameters can aid the clinical diagnosis of Zika virus and DENV infections in the early stages.

Technical Appendix Baseline demographic variables for patients in cohorts suspected to have Zika virus infection or dengue virus infection, Singapore; changes in posttest probability of Zika and dengue virus infections based on presence or absence of selected clinical and laboratory parameters within the first 5 days after symptom onset.
