# Supplementary material for: Diagnostic Accuracy of Parameters for Zika and Dengue Virus Infections, Singapore
Source: Emerg Infect Dis. 2017 Dec;23(12):2085–8. doi: 10.3201/eid2312.171224 (PMC5708236; doi:10.3201/eid2312.171224)
Supplement: Technical Appendix — Baseline demographic variables for patients in cohorts suspected to have Zika virus infection or dengue virus infection, Singapore; changes in posttest probability of Zika and dengue virus infections based on presence or absence of selected clinical and laboratory parameters within the first 5 days after symptom onset. [file 17-1224-Techapp-s1.pdf]

Article DOI: https://doi.org/10.3201/eid2312.171224

Diagnostic Accuracy of Parameters for Zika and Dengue Virus Infections, Singapore

Technical Appendix

Technical Appendix Table 1. Baseline demographic data of patients in cohorts suspected to have Zika and dengue virus infection, Singapore*

Demographic variable	Zika cohort, n = 281	Dengue cohort, n = 310
Mean age, y (± SD)	34.9 (13.0)	35.8 (11.0)
Median age, y (IQR)	32 (26–43)	34 (27–42)
Male sex, no. (%)	178 (63.3)	249 (80.3)
Diabetes mellitus, no. (%)	11 (3.9)	2 (0.6)
Cardiovascular disease, no. (%)	4 (1.4)	1 (0.3)
Cerebrovascular disease, no. (%)	4 (1.4)	1 (0.3)
Charlson's Comorbidity Index score >3	1 (0.4)	0

*IQR, interquartile range.

Technical Appendix Table 2. Changes in posttest probability of Zika and dengue virus infections based on presence or absence of selected clinical and laboratory parameters within the first 5 days of symptom onset, Singapore*†

Parameter(s)	Zika virus infection	Dengue virus infection
Rash	↑ /↓↓↓	0
Leukopenia <3.6 × 10^9/L	↑	↑ /↓↓
Lymphopenia <0.9 × 10^9/L	0	↑/↓
Any GI* symptom and lymphopenia	0	↑/↓
Rash and conjunctivitis	↑↑↑	0
Documented fever and rash	↑	0
Documented fever and rash and any GI symptom	↑	↑
Documented fever and rash and lymphopenia	0	↑
Documented fever and thrombocytopenia	0	↑
Documented fever and lymphopenia	0	↑
Documented fever and lymphopenia and thrombocytopenia	0	↑↑

*GI, gastrointestinal, i.e., nausea, vomiting, diarrhea, and abdominal pain. Up arrows signs indicate increased probability of infection if parameter(s) is/are present, as follows: ↑, +15%–24%; ↑↑, +25%–34%; ↑↑↑, +35%–44%. Down arrows indicate decreased probability of infection if parameter(s) is/are absent: ↓, −15%–24%; ↓↓, −25%–34%; ↓↓↓, −35%–44.
†McGee S. Simplifying likelihood ratios. J Gen Intern Med. 2002;17:646–9.
